# Chronic Inflammation and Hearing Loss: Key Biomarkers and Subgroup Differences by Gender and BMI in a National Cohort

**DOI:** 10.1002/iid3.70188

**Published:** 2025-04-09

**Authors:** Zhe Peng, Chun‐li Zhao, Guo‐peng Wang, Qian Wu, Shu‐sheng Gong

**Affiliations:** ^1^ Department of Otolaryngology‐Head and Neck Surgery, Beijing Friendship Hospital Capital Medical University Beijing China; ^2^ Clinical Center for Hearing Loss Capital Medical University Beijing China

**Keywords:** hearing loss, inflammatory biomarkers, NHANES, population‐based study, systemic immune‐inflammation index

## Abstract

**Background:**

Hearing loss (HL) significantly impacts quality of life and economic status worldwide. Chronic inflammation is suggested to influence hearing, yet the connection with inflammation‐related indexes in the general population is not well understood.

**Methods:**

This cross‐sectional study analyzed data from 7231 adults from six cycles (2005–2012 and 2015–2018) of the National Health and Nutrition Examination Survey (NHANES). It examined the correlation between systemic immune‐inflammatory biomarkers (NLR, SII, PLR, and LMR) and auditory threshold shifts/HL using multivariable logistic regression models. Smooth curve fitting visualized the association, and log‐likelihood ratio tests determined the existence of thresholds in biomarker effects, supplemented by subgroup analyses.

**Results:**

After adjustments, significant associations were found for low‐frequency HL with ln‐transformed NLR (OR = 1.29, 95% CI: 1.06–1.56, *p* = 0.0116), ln‐SII (OR = 1.31, 95% CI: 1.08–1.59, *p* = 0.0065), and ln‐LMR (OR = 0.74, 95% CI: 0.60–0.91, *p* = 0.00043). For high‐frequency HL, similar patterns were observed for ln‐SII (OR = 1.25, 95% CI: 1.05–1.48, *p* = 0.0105) and ln‐LMR (OR = 0.76, 95% CI: 0.64–0.90, *p* = 0.007); however, the association with ln‐NLR did not reach statistical significance (OR = 1.18, 95% CI: 1.00–1.40, *p* = 0.0562). NLR and SII positively correlated with HL, while LMR showed a negative correlation. No significant association was noted with PLR. Dose–response relationships were observed, particularly between LMR and all categorized frequencies of HL and between SII and high‐frequency HL. Subgroup analyses indicated that NLR and SII are risk factors for HL in healthy BMI males, with LMR being more protective in males, the elderly, and diabetics.

**Conclusions:**

Systemic inflammation‐related indexes, especially SII, are predictive of both high‐ and low‐frequency HL, highlighting the role of inflammatory homeostasis in hearing health. LMR may offer protective effects, particularly in specific subgroups. These findings suggest potential targets for HL treatment by regulating inflammation, warranting further investigation into their clinical application.

## Graphical abstract

The study highlights the role of inflammatory homeostasis in hearing health. LMR may offer protective effects, particularly in male, old, and diabetes subgroups. These findings suggest potential targets for hearing loss treatment by regulating inflammation.

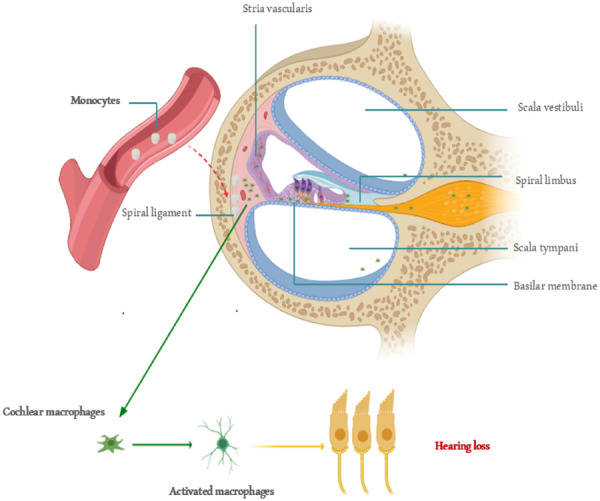

## 1 Introduction

Hearing loss (HL) is the fourth leading cause of disability worldwide. According to data from the World Health Organization (WHO), approximately 1.5 billion people globally are affected by HL [1]. This number is expected to increase due to the aging population [2]. It is estimated that by 2050, nearly 2.5 billion people will have some degree of HL, with at least 700 million requiring rehabilitation services [3]. Furthermore, HL is considered one of the most clinically significant risk factors for cognitive decline and dementia [4], but it is also regarded as one of the modifiable risk factors [5]. Therefore, early detection and prevention of HL are of great importance. The etiology of HL is complex and multifactorial, with numerous contributing factors. These causes include congenital factors, infections, noise exposure, aging, trauma, and immune‐related conditions [6].

Recent research has increasingly suggested that inflammation may affect cochlear function [7, 8, 9, 10]. Some studies suggest that chronic inflammation could be a new target for future treatments of age‐related HL [8, 9]. Also, inflammatory responses induced by traumatic and ototoxic insults may exacerbate cochlear pathology, contributing to both acute and chronic cellular damage within the cochlea [11]. Inflammation is a complicated physiologic reaction that attempts to repair damaged tissue. It is a dynamic process incorporating cells, serum components, and cellular products [12]. Although the inflammatory response is a normal physiological process that facilitates tissue repair, uncontrolled progression can cause tissue damage. New laboratory indices are developed to provide insight into inflammatory status. Consequently, there is growing interest in identifying biomarkers that can assess systemic inflammation associated with disease or have significant implications for disease progression and prognosis [13, 14, 15].

Regarding inflammatory markers associated with HL, C‐reactive protein (CRP) [7, 8, 16], serum glycoprotein A (GlycA) [17], interleukin‐6 (IL‐6) [18, 19], interleukin‐1β (IL‐1β) [20], tumor necrosis factor‐α (TNF‐α) [18], white blood cell count [21, 22], and neutrophil count [19, 21] are associated with HL [9, 23]. Several composite inflammatory indices, such as the neutrophil‐to‐lymphocyte ratio (NLR), Systemic Immune‐Inflammation Index (SII), lymphocyte‐to‐monocyte ratio (LMR), and platelet‐to‐lymphocyte ratio (PLR), have been recognized as prognostic indicators for various cancers and inflammatory diseases [14]. Through different focal points, these inflammatory markers combine the analysis of peripheral lymphocytes, platelets, neutrophils, and monocyte counts to obtain corresponding index values. Compared to a single inflammatory marker, this approach can comprehensively reflect local immune status and systemic inflammatory conditions. The NLR and PLR have been used to investigate the relationship between sudden sensorineural HL (ISSNHL) [24, 25], and the NLR has been considered a quick and reliable indicator for predicting the diagnosis and the prognosis of ISSNHL [26] and HL in diabetic patients [27]. The SII is also considered to be associated with HL [28], and as a novel index, the SII can be an indicator of ISSNHL and predict its prognosis [29].

However, current studies are predominantly single‐center or focus primarily on a single inflammatory indicator, and there is a lack of nationwide investigation on the correlation between these important composite inflammatory indices and HL. Single‐marker studies, while informative, often lack the comprehensiveness to fully assess the complex interplay between systemic inflammation and HL. By incorporating multiple markers such as NLR, SII, PLR, and LMR, we aim to provide a more holistic view of the inflammatory processes that may contribute to HL at a population level. This broader approach is necessary to improve the clinical applicability of inflammatory indices in predicting HL across diverse populations. Therefore, it is still challenging to assess the applicability of these indices for population‐wide implementation. This study aims to evaluate the relationship between the levels of NLR, SII, PLR, and LMR and the prevalence of HL.

## 2 Methods

### 2.1 Study Design and Population

This study employed a cross‐sectional design, utilizing data from the National Health and Nutrition Examination Survey (NHANES), conducted between 2005–2012 and 2015–2018. NHANES is a national survey targeting the noninstitutionalized civilian population of the United States. The survey is conducted biannually, with rigorous sampling and data collection procedures previously documented in detail. NHANES is conducted by the National Center for Health Statistics (NCHS) of the Centers for Disease Control and Prevention (CDC), and its protocols have been approved by the NHANES Institutional Review Board [30].

### 2.2 Inclusion and Exclusion Criteria

Adults (aged > 19 years) with complete hearing test data were included in the study.

The following exclusion criteria were applied:

1. Those who were aged ≤ 19 years.
2. Participants without NLR, SII, PLR, and LMR data.
3. Participants without complete data about audiometrics.
4. Participants with abnormal findings in ear examinations were not eligible for inclusion, such as cases with ear tubes, infections, abnormal otoscopic findings, cerumen blockage, or substandard tympanometry results.
5. Participants lacked completed essential covariates such as Body Mass Index (BMI), Poverty Income Ratio (PIR), hypertension, and diabetes.

### 2.3 Definition of Inflammatory‐Related Indicators

All test parameters were initially recorded using a Beckman Coulter MAXM automated analytical instrument. Neutrophil, monocyte, platelet, and lymphocyte counts were reported as ×10^3^ cells/μL. Considering future clinical feasibility, applicability, effectiveness, and the body's comprehensive immune and inflammatory response, four indicators related to systemic inflammation were analyzed: NLR, SII, PLR, and LMR.

The calculation formulas for NLR, SII, PLR, and LMR are as follows [13, 14, 31]:

NLR = Neutrophil count/Lymphocyte count

SII = (Neutrophil count × Platelet counts)/Lymphocyte count

PLR = Platelet counts/Lymphocyte count

LMR = Lymphocyte count/Monocyte count

### 2.4 Hearing Assessment and Definition of HL

All Audiometry Component sections were performed by a trained examiner on examinees in a dedicated, sound‐isolating room in the mobile examination center (MEC). Instrumentation for the Audiometry Component included an Interacoustics Model AD226 audiometer with standard TDH‐39 headphones and Etymotic EarTone 3A insert earphones. Tympanometry was performed using a Micro Audiometrics Earscan Acoustic Impedance Tympanometer. In an acoustically controlled environment, experienced audiologists performed pure tone audiometry (PTA) to assess hearing. Testing covered frequencies from 0.5 to 8 kHz, with duplicate tests at 1 kHz to ensure reliability. HL was defined as thresholds greater than and equal to 25 dB at any categorized frequency, according to the recommendations of the WHO (2021) [3]. The results were categorized into low‐frequency (0.5, 1, and 2 kHz) [28, 32, 33], speech‐frequency (0.5, 1, 2, and 4 kHz) [28, 34, 35], and high‐frequency (4, 6, and 8 kHz) hearing thresholds [28, 32].

### 2.5 Other Variables of Interest

Potential covariates that could confound the relationship between inflammatory‐related indicators and auditory health were accounted for in a multivariate‐adjusted model. These covariates included age, gender, race, PIR, BMI, hypertension, and diabetes.

Demographic details, including gender, age, race, PIR, hypertension, and diabetes status, were obtained using a standardized questionnaire. Considering the impact of age on hearing, the population was divided into two groups for subgroup analysis: < 60 years and ≥ 60 years. In other analyses, age was treated as a continuous variable. Physical examinations were used to obtain BMI data, which was divided into three categories: normal weight (< 25 kg/m^2^), overweight (≥ 25 kg/m^2^ and ≤ 30 kg/m^2^), and obese (≥ 30 kg/m^2^). Household income levels were determined using the self‐reported family PIR. Hypertension was defined as a self‐reported high blood pressure diagnosis. Participants who answered “yes” to either condition were classified as having hypertension. Diabetes was defined as either a self‐reported medical diagnosis or the use of antihyperglycemic medications. Participants who answered “yes” or “borderline” to either question were classified as having diabetes [32, 36].

### 2.6 Statistical Analysis

The analyses were conducted using EmpowerStats (version 4.2) and R (version 3.4.3). Data from the 2005–2012 and 2015–2018 NHANES cycles were combined, and 12‐year sampling weights were created using the 2‐year sampling weights (WTMEC2YR) provided by NHANES. These weights accounted for oversampling, survey nonresponse, and post‐stratification inherent in the complex survey design. All analyses followed the NCHS analytic guidelines to account for the complex survey design [37].

Continuous variables were presented as mean ± SD, while categorical variables were expressed as numbers (percentages). A Student's *t*‐test or ANOVA was used to compare demographics, with the *χ* ^2^ test employed for categorical variables. Logistic regression models were used to estimate the *β*/odds ratios (ORs) and 95% confidence intervals (CIs) for the association between NLR, SII, PLR, and LMR levels (including tertiles) and the hearing thresholds/prevalence of HL at various frequencies. Model 1 was unadjusted. Model 2 was adjusted for age, gender, and race. In the fully adjusted model (Model 3), covariates including age, gender, race, family income–poverty ratio, BMI, hypertension, and diabetes were adjusted. The study performed a smooth curve fitting analysis to better visualize their associations. We first use smooth curve fitting to examine whether the independent variable is partitioned into intervals. Then, we apply a log‐likelihood ratio test to determine if there is a threshold effect.

The study performed subgroup analyses based on clinical characteristics such as gender (male/female), age (under 60 years or 60 years and older), BMI (less than 25 kg/m^2^, 25–30 kg/m^2^, and 30 kg/m^2^ or more), hypertension (yes/no), and diabetes (yes/no). Furthermore, the study analyzed the *p* values for interactions within these groups.

## 3 Results

### 3.1 Characteristics of Participation

**Figure 1:**
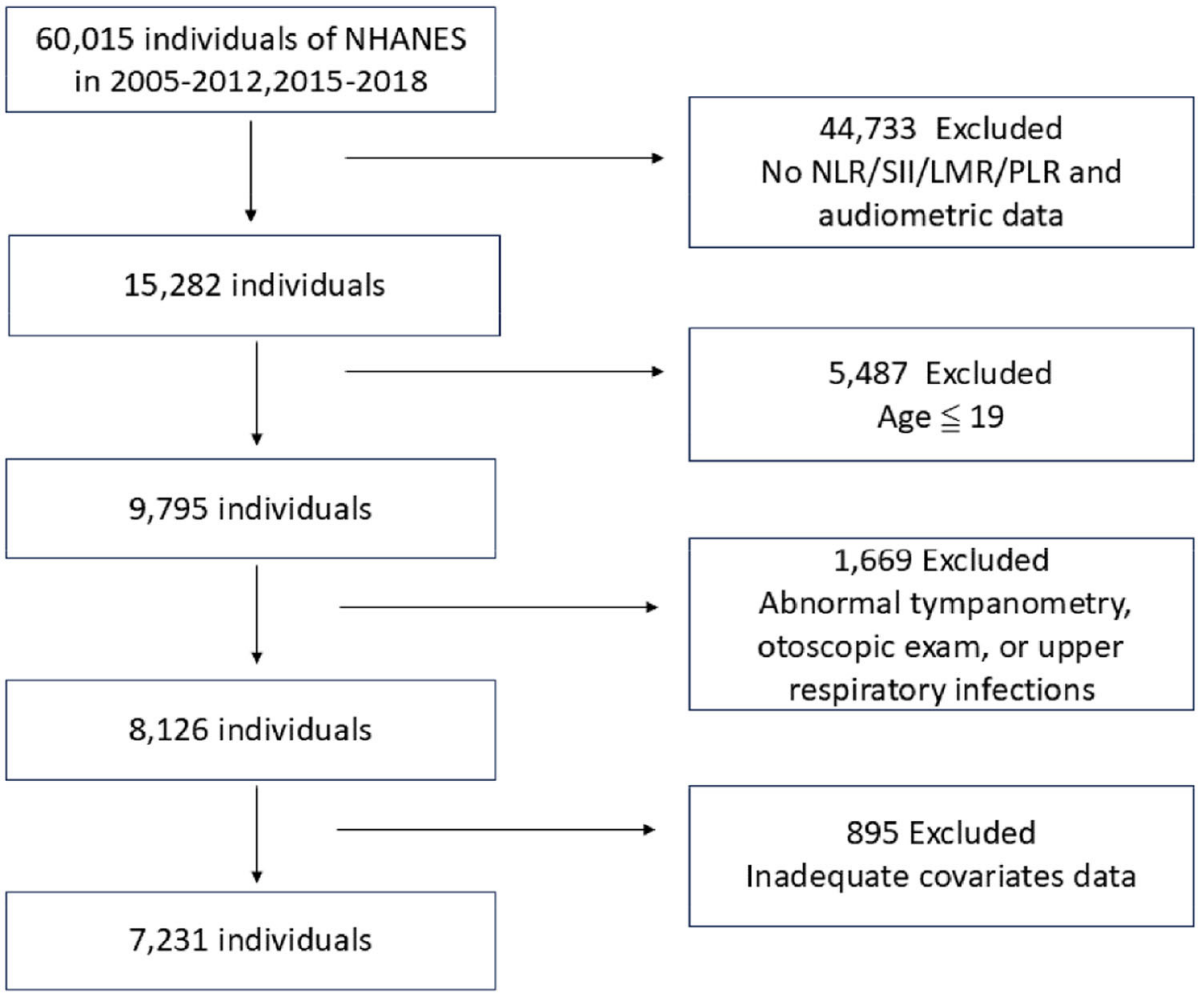
Flowchart of participant selection. From 60,015 NHANES participants (2005–2012, 2015–2018), 44,733 were excluded for missing NLR/SII/LMR/PLR and audiometric data, 5487 were under age 19, 1669 had abnormal tympanometry, otoscopic exam, or upper respiratory infections, and 895 had inadequate covariate data, resulting in a final sample of 7231 participants.

From 2005 to 2012 and from 2015 to 2018, a total of 60,015 individuals participated in the NHANES survey. Among these, 44,733 were excluded due to missing exposure and outcome indicators for the study. Of the remaining 15,282 individuals, 5487 were excluded because they were 19 years old or younger. Out of the remaining 9795 participants, 1669 were further excluded for the following reasons: (1) 29 participants had tympanostomy tubes, (2) 1009 participants had upper respiratory infections during the survey, and (3) 631 participants had invalid or indeterminate tympanogram in one or both ears. Among the remaining 8126 participants, 895 were further excluded due to missing key covariates: (1) 806 participants lacked PIR data, (2) 76 participants lacked BMI data, (3) 12 participants lacked hypertension data, and (4) 1 participant lacked diabetes data. Finally, 7231 participants were included (Figure 1).

As shown in Table 1, individuals with HL at specific frequencies tend to have a higher average age compared to those without HL at these frequencies. Specifically, for individuals with low‐frequency HL, the average age is 69.68 years, compared to 45.94 years for those without low‐frequency HL. Similarly, for speech‐frequency HL, individuals with HL have an average age of 66.73 years, while those without it average 41.19 years. For high‐frequency HL, the average age is 63.27 years for individuals with HL, compared to 38.17 years for those without HL. These findings indicate that high‐frequency HL tends to occur at a younger age than low‐ and speech‐frequency HL. Compared to individuals without HL, those with HL have higher BMI values, with *p* values < 0.05 in the speech‐frequency and high‐frequency HL groups. Additionally, the percentage of individuals with hypertension and diabetes is higher in the HL groups than in the non‐HL groups.

**Table 1:** Baseline characteristics of participants weighted based on hearing loss (HL).

Variable	Low‐frequency HL			Speech‐frequency HL			High‐frequency HL		
	< 25 (dB)	≥ 25 (dB)	*p* value	< 25 (dB)	≥ 25 (dB)	*p* value	< 25 (dB)	≥ 25 (dB)	*p* value
*N*	6142	1089		4875	2356		3964	3267	
Continuous variables, mean ± SD									
Age (years)	45.94 ± 16.62	69.68 ± 12.59	< 0.001	41.19 ± 14.30	66.73 ± 12.34	< 0.001	38.17 ± 12.81	63.27 ± 13.68	< 0.001
PIR	2.54 ± 1.63	2.39 ± 1.49	0.057	2.54 ± 1.65	2.46 ± 1.54	0.296	2.55 ± 1.65	2.47 ± 1.57	0.119
NLR	2.09 ± 1.10	2.44 ± 1.30	< 0.001	2.01 ± 0.97	2.40 ± 1.39	< 0.001	2.00 ± 0.94	2.31 ± 1.32	< 0.001
PLR	121.30 ± 48.09	131.04 ± 57.97	< 0.001	119.59 ± 43.84	129.34 ± 59.81	< 0.001	119.25 ± 43.44	127.04 ± 56.31	< 0.001
LMR	4.37 ± 2.38	3.59 ± 1.60	< 0.001	4.53 ± 2.52	3.68 ± 1.60	< 0.001	4.58 ± 2.68	3.85 ± 1.64	< 0.001
SII	506.00 ± 309.78	567.21 ± 350.11	< 0.001	494.85 ± 282.60	557.36 ± 374.72	< 0.001	493.23 ± 276.31	541.89 ± 358.32	< 0.001
BMI (kg/m^2^)	29.19 ± 7.06	29.10 ± 6.22	0.340	29.14 ± 7.19	29.25 ± 6.39	0.010	28.99 ± 7.30	29.39 ± 6.47	< 0.001
Categorical variables, %									
Gender *N* (%)			0.166			< 0.001			< 0.001
Male	2996 (48.78%)	556 (51.06%)		2209 (45.31%)	1343 (57.00%)		1734 (43.74%)	1818 (55.65%)	
Female	3146 (51.22%)	533 (48.94%)		2666 (54.69%)	1013 (43.00%)		2230 (56.26%)	1449 (44.35%)	
Race *N* (%)			< 0.001			< 0.001			< 0.001
Mexican American	846 (13.77%)	132 (12.12%)		699 (14.34%)	279 (11.84%)		566 (14.28%)	412 (12.61%)	
Other Hispanic	689 (11.22%)	83 (7.62%)		567 (11.63%)	205 (8.70%)		465 (11.73%)	307 (9.40%)	
Non‐Hispanic White	2230 (36.31%)	612 (56.20%)		1593 (32.68%)	1249 (53.01%)		1259 (31.76%)	1583 (48.45%)	
Non‐Hispanic Black	1409 (22.94%)	172 (15.79%)		1194 (24.49%)	387 (16.43%)		974 (24.57%)	607 (18.58%)	
Other race	968 (15.76%)	90 (8.26%)		822 (16.86%)	236 (10.02%)		700 (17.66%)	358 (10.96%)	
Education *N* (%)			< 0.001			< 0.001			< 0.001
Less than 9th Grade	459 (7.47%)	194 (17.81%)		308 (6.32%)	345 (14.64%)		221 (5.58%)	432 (13.22%)	
9–11th Grade	716 (11.66%)	185 (16.99%)		527 (10.81%)	374 (15.87%)		396 (9.99%)	505 (15.46%)	
High school grad/GED or equivalent	1340 (21.82%)	264 (24.24%)		1008 (20.68%)	596 (25.30%)		797 (20.11%)	807 (24.70%)	
Some college or AA degree	1920 (31.26%)	259 (23.78%)		1584 (32.49%)	595 (25.25%)		1327 (33.48%)	852 (26.08%)	
College graduate or above	1706 (27.78%)	186 (17.08%)		1448 (29.70%)	444 (18.85%)		1223 (30.85%)	669 (20.48%)	
Don't know	1 (0.02%)	1 (0.09%)		0 (0.00%)	2 (0.08%)		0 (0.00%)	2 (0.06%)	
Marital status *N* (%)			< 0.001			< 0.001			< 0.001
Married	3097 (50.42%)	592 (54.36%)		2359 (48.39%)	1330 (56.45%)		1856 (46.82%)	1833 (56.11%)	
Widowed	344 (5.60%)	269 (24.70%)		161 (3.30%)	452 (19.19%)		86 (2.17%)	527 (16.13%)	
Divorced	591 (9.62%)	105 (9.64%)		431 (8.84%)	265 (11.25%)		312 (7.87%)	384 (11.75%)	
Separated	217 (3.53%)	29 (2.66%)		179 (3.67%)	67 (2.84%)		130 (3.28%)	116 (3.55%)	
Never married	1299 (21.15%)	65 (5.97%)		1201 (24.64%)	163 (6.92%)		1097 (27.67%)	267 (8.17%)	
Living with partner	593 (9.65%)	29 (2.66%)		543 (11.14%)	79 (3.35%)		482 (12.16%)	140 (4.29%)	
Hypertension *N* (%)			< 0.001			< 0.001			< 0.001
Yes	1976 (32.17%)	634 (58.22%)		1290 (26.46%)	1320 (56.03%)		875 (22.07%)	1735 (53.11%)	
No	4166 (67.83%)	455 (41.78%)		3585 (73.54%)	1036 (43.97%)		3089 (77.93%)	1532 (46.89%)	
Diabetes *N* (%)			< 0.001			< 0.001			< 0.001
Yes	708 (11.53%)	257 (23.60%)		425 (8.72%)	540 (22.92%)		278 (7.01%)	687 (21.03%)	
No	5434 (88.47%)	832 (76.40%)		4450 (91.28%)	1816 (77.08%)		3686 (92.99%)	2580 (78.97%)	

### 3.2 Associations Between NLR, SII, PLR, and LMR Index and Auditory Threshold Shifts

To analyze the relationship between NLR, SII, PLR, and LMR with hearing thresholds, multivariate logistic regression was used. In the unadjusted model, NLR, SII, and PLR were positively associated with elevated speech‐frequency and high‐frequency thresholds, while LMR showed a negative association. Low‐frequency PTA (*β* values: 3.76, 1.99, 2.06, and −6.74, respectively), speech‐frequency PTA (*β* values: 6.07, 2.90, 3.25, and −11.4, respectively), and high‐frequency PTA (*β* values: 8.39, 3.81, 4.45, and −16.05, respectively) (Table 2.1).

**Table 2.1:** Logistic regression analysis between NLR/SII/PLR/LMP index with hearing‐frequency prevalence.

Frequency/Index	*β* (95% CI), *p* value of PTA levels, dB		
	Non‐adjusted	Adjust I	Adjust II
Low‐frequency PTA			
NLR	3.76 (3.15, 4.36) < 0.0001	1.39 (0.90, 1.89) < 0.0001	1.30 (0.81, 1.79) < 0.0001
SII	1.99 (1.47, 2.50) < 0.0001	1.08 (0.67, 1.50) < 0.0001	0.99 (0.58, 1.41) < 0.0001
PLR	2.06 (1.32, 2.80) < 0.0001	0.30 (−0.29, 0.88) 0.3202	0.54 (−0.05, 1.12) 0.0710
LMR	−6.74 (−7.44, −6.05) < 0.0001	−1.81 (−2.41, −1.21) < 0.0001	−1.83 (−2.42, −1.24) < 0.0001
Speech‐frequency PTA			
NLR	6.07 (5.27, 6.88) < 0.0001	1.81 (1.26, 2.35) < 0.0001	1.71 (1.17, 2.25) < 0.0001
SII	2.90 (2.21, 3.59) < 0.0001	1.41 (0.95, 1.87) < 0.0001	1.32 (0.86, 1.78) < 0.0001
PLR	3.25 (2.26, 4.24) < 0.0001	0.67 (0.02, 1.32) 0.0428	0.95 (0.31, 1.59) 0.0038
LMR	−11.40 (−12.32, −10.48) < 0.0001	−2.34 (−3.00, −1.68) < 0.0001	−2.37 (−3.02, −1.72) < 0.0001
High‐frequency PTA			
NLR	8.39 (7.29, 9.49) < 0.0001	2.22 (1.49, 2.94) < 0.0001	2.12 (1.40, 2.84) < 0.0001
SII	3.81 (2.87, 4.76) < 0.0001	1.73 (1.12, 2.35) < 0.0001	1.64 (1.04, 2.25) < 0.0001
PLR	4.45 (3.10, 5.81) < 0.0001	1.04 (0.18, 1.91) 0.0179	1.36 (0.50, 2.22) 0.0019
LMR	−16.05 (−17.31, −14.80) < 0.0001	−2.87 (−3.75, −1.99) < 0.0001	−2.91 (−3.78, −2.04) < 0.0001

*Note:* Model 1: No adjustment for co‐variables. Model 2: Adjusted for age, gender, and race. Model 3: Adjusted for age, gender, race, BMI, PIR, hypertension, and diabetes.

After adjusting for age, gender, race, PIR, BMI, hypertension, and diabetes, NLR, SII, and PLR were still positively associated with elevated speech‐frequency and high‐frequency thresholds, while LMR was negatively associated with speech‐frequency PTA (*β* values: 1.71, 1.32, 0.95, and −2.37, respectively) and high‐frequency PTA (*β* values: 2.12, 1.64, 1.36, and −2.91, respectively) (Table 2.1). Interestingly, for low‐frequency PTA, only NLR, SII, and LMR were significantly associated with elevated thresholds (*β* values: 1.30, 0.99, and −1.83, respectively). PLR was not significantly associated with low‐frequency thresholds.

### 3.3 Associations Between NLR, SII, PLR, and LMR Index and HL

To further analyze the relationship between NLR, SII, PLR, and LMR with HL, PTA thresholds were converted into binary variables, with PTA < 25 dB defined as no HL and PTA ≥ 25 dB defined as HL. Multivariate logistic regression was then used. The values of all inflammatory indices were In‐transformed before conducting the multiple regression analysis.

After adjusting for age, gender, race, PIR, BMI, hypertension, and diabetes, NLR, SII, and LMR were significantly associated with HL at all categorized frequencies (low, speech, and high). NLR and SII were positively associated, while LMR was negatively associated. For low‐frequency HL, the ORs were 1.22 (95% CI: 1.04, 1.45) for NLR, 1.17 (95% CI: 1.02, 1.34) for SII, and 0.78 (95% CI: 0.64, 0.96) for LMR. For speech‐frequency HL, the ORs were 1.28 (95% CI: 1.09, 1.50) for NLR, 1.20 (95% CI: 1.05, 1.37) for SII, and 0.65 (95% CI: 0.53, 0.79) for LMR. For high‐frequency HL, the ORs were 1.29 (95% CI: 1.10, 1.51) for NLR, 1.21 (95% CI: 1.06, 1.39) for SII, and 0.73 (95% CI: 0.60, 0.88) for LMR (Table 2.2). However, PLR was not significantly associated with HL at any frequency.

**Table 2.2:** Logistic regression analysis between NLR/SII/PLR/LMP index with low/speech/high‐frequency hearing loss (HL) (In‐transformed of the index).

Frequency/Index	OR (95% CI), *p* value of HL		
	Non‐adjusted	Adjust I	Adjust II
Low‐frequency HL			
NLR	2.06 (1.79, 2.38) < 0.0001	1.24 (1.06, 1.47) 0.0090	1.22 (1.04, 1.45) 0.0163
NLR Tertile			
Low	1.0	1.0	1.0
Middle	1.40 (1.18, 1.66) 0.0001	1.31 (1.07, 1.61) 0.0079	1.31 (1.07, 1.61) 0.0082
High	2.06 (1.75, 2.43) < 0.0001	1.31 (1.08, 1.59) 0.0058	1.29 (1.06, 1.56) 0.0116
*p* for trend	< 0.0001	0.0089	0.0180
SII	1.44 (1.28, 1.63) < 0.0001	1.18 (1.03, 1.36) 0.0175	1.17 (1.02, 1.34) 0.0273
SII Tertile			
Low	1.0	1.0	1.0
Middle	1.40 (1.19, 1.65) < 0.0001	1.40 (1.16, 1.70) 0.0006	1.41 (1.16, 1.71) 0.0006
High	1.65 (1.41, 1.95) < 0.0001	1.32 (1.09, 1.60) 0.0046	1.31 (1.08, 1.59) 0.0065
*p* for trend	< 0.0001	0.0066	0.0092
PLR	1.54 (1.30, 1.84) < 0.0001	1.04 (0.87, 1.25) 0.6534	1.09 (0.91, 1.32) 0.3596
PLR Tertile			
Low	1.0	1.0	1.0
Middle	1.05 (0.89, 1.23) 0.5969	1.04 (0.86, 1.26) 0.7045	1.07 (0.88, 1.30) 0.5006
High	1.46 (1.24, 1.70) < 0.0001	1.05 (0.87, 1.26) 0.6120	1.10 (0.91, 1.33) 0.3185
*p* for trend	< 0.0001	0.6172	0.3228
LMR	0.26 (0.22, 0.31) < 0.0001	0.79 (0.64, 0.96) 0.0191	0.78 (0.64, 0.96) 0.0173
LMR Tertile			
Low	1.0	1.0	1.0
Middle	0.48 (0.41, 0.55) < 0.0001	0.88 (0.74, 1.06) 0.1840	0.90 (0.75, 1.08) 0.2676
High	0.30 (0.25, 0.35) < 0.0001	0.74 (0.60, 0.91) 0.0047	0.74 (0.60, 0.91) 0.0043
*p* for trend	< 0.0001	0.0048	0.0050
Speech‐frequency HL			
NLR	2.08 (1.86, 2.33) < 0.0001	1.31 (1.12, 1.54) 0.0007	1.28 (1.09, 1.50) 0.0025
NLR Tertile			
Low	1.0	1.0	1.0
Middle	1.22 (1.08, 1.39) 0.0018	1.11 (0.93, 1.32) 0.2535	1.10 (0.92, 1.31) 0.2813
High	1.92 (1.70, 2.17) < 0.0001	1.21 (1.02, 1.44) 0.0325	1.16 (0.98, 1.39) 0.0917
*p* for trend	< 0.0001	0.0325	0.0924
SII	1.36 (1.24, 1.50) < 0.0001	1.22 (1.07, 1.40) 0.0028	1.20 (1.05, 1.37) 0.0078
SII Tertile			
Low	1.0	1.0	1.0
Middle	1.19 (1.05, 1.35) 0.0054	1.21 (1.02, 1.43) 0.0305	1.20 (1.01, 1.42) 0.0429
High	1.43 (1.27, 1.62) < 0.0001	1.19 (1.00, 1.42) 0.0483	1.16 (0.97, 1.38) 0.1026
*p* for trend	< 0.0001	0.0465	0.0996
PLR	1.48 (1.29, 1.69) < 0.0001	1.06 (0.88, 1.27) 0.5543	1.15 (0.96, 1.39) 0.1268
PLR Tertile			
Low	1.0	1.0	1.0
Middle	1.02 (0.90, 1.15) 0.7484	1.07 (0.90, 1.27) 0.4343	1.13 (0.95, 1.35) 0.1595
High	1.43 (1.27, 1.61) < 0.0001	1.08 (0.91, 1.28) 0.3823	1.18 (0.99, 1.40) 0.0671
*p* for trend	< 0.0001	0.3828	0.0673
LMR	0.21 (0.19, 0.25) < 0.0001	0.66 (0.55, 0.80) < 0.0001	0.65 (0.53, 0.79) < 0.0001
LMR Tertile			
Low	1.0	1.0	1.0
Middle	0.41 (0.36, 0.46) < 0.0001	0.72 (0.61, 0.86) 0.0002	0.74 (0.62, 0.88) 0.0006
High	0.28 (0.24, 0.31) < 0.0001	0.68 (0.57, 0.82) < 0.0001	0.67 (0.56, 0.81) < 0.0001
*p* for trend	< 0.0001	< 0.0001	< 0.0001
High‐frequency HL			
NLR	1.84 (1.66, 2.04) < 0.0001	1.32 (1.13, 1.54) 0.0004	1.29 (1.10, 1.51) 0.0014
NLR Tertile			
Low	1.0	1.0	1.0
Middle	1.13 (1.01, 1.27) 0.0395	1.04 (0.88, 1.23) 0.6309	1.04 (0.88, 1.23) 0.6181
High	1.73 (1.54, 1.94) < 0.0001	1.22 (1.03, 1.44) 0.0212	1.18 (1.00, 1.40) 0.0562
*p* for trend	< 0.0001	0.0223	0.0580
SII	1.27 (1.17, 1.39) < 0.0001	1.24 (1.09, 1.42) 0.0010	1.21 (1.06, 1.39) 0.0045
SII Tertile			
Low	1.0	1.0	1.0
Middle	1.11 (0.99, 1.24) 0.0713	1.13 (0.96, 1.33) 0.1427	1.12 (0.95, 1.32) 0.1785
High	1.36 (1.21, 1.52) < 0.0001	1.29 (1.09, 1.53) 0.0027	1.25 (1.05, 1.48) 0.0105
*p* for trend	< 0.0001	0.0027	0.0104
PLR	1.37 (1.21, 1.56) < 0.0001	1.08 (0.90, 1.30) 0.3974	1.19 (0.99, 1.43) 0.0630
PLR Tertile			
Low	1.0	1.0	1.0
Middle	0.95 (0.85, 1.06) 0.3622	0.97 (0.83, 1.15) 0.7594	1.04 (0.88, 1.23) 0.6515
High	1.30 (1.16, 1.45) < 0.0001	1.00 (0.85, 1.18) 0.9701	1.08 (0.92, 1.28) 0.3552
*p* for trend	< 0.0001	0.9645	0.3553
LMR	0.28 (0.25, 0.32) < 0.0001	0.75 (0.62, 0.90) 0.0018	0.73 (0.60, 0.88) 0.0009
LMR Tertile			
Low	1.0	1.0	1.0
Middle	0.45 (0.40, 0.51) < 0.0001	0.77 (0.65, 0.91) 0.0022	0.78 (0.66, 0.92) 0.0043
High	0.34 (0.30, 0.38) < 0.0001	0.77 (0.65, 0.91) 0.0024	0.76 (0.64, 0.90) 0.0017
*p* for trend	< 0.0001	0.0030	0.0021

*Note:* Model 1: No adjustment for co‐variables. Model 2: Adjusted for age, gender, and race. Model 3: Adjusted for age, gender, race, BMI, PIR, hypertension, and diabetes.

To improve the reliability of our outcomes and further evaluate the association of these indicators with HL at different levels, we divided the NLR, SII, PLR, and LMR scores into tertiles to examine their association with HL at various frequencies. In low‐frequency HL, NLR, SII, and LMR in the highest tertile showed significant associations, with NLR and SII being positively correlated and LMR being negatively correlated. For NLR, the OR in the highest tertile was 1.29 (95% CI: 1.06, 1.56), and for SII, the OR was 1.31 (95% CI: 1.08, 1.59). LMR had an OR of 0.74 (95% CI: 0.60, 0.91) in the highest tertile. Notably, only LMR showed an increasing effect size with higher tertiles (*p* for trend: 0.005).

In speech‐frequency HL, only LMR maintained a significant negative association in the highest tertile, with an OR of 0.65 (95% CI: 0.53, 0.79), and the effect size peaked in this tertile (*p* for trend: < 0.0001). In high‐frequency HL, both SII and LMR in the highest tertile showed significant associations, with SII being positively correlated (OR: 1.25, 95% CI: 1.05, 1.48) and LMR being negatively correlated (OR: 0.76, 95% CI: 0.64, 0.90). The effect size for LMR continued to increase robustly with higher tertiles (*p* for trend: 0.0021). PLR in the highest tertile did not show significant associations with HL at any frequency (Table 2.2).

### 3.4 Relationship Between NLR, SII, PLR, and LMR Index and HL Visualization

**Figure 2:**
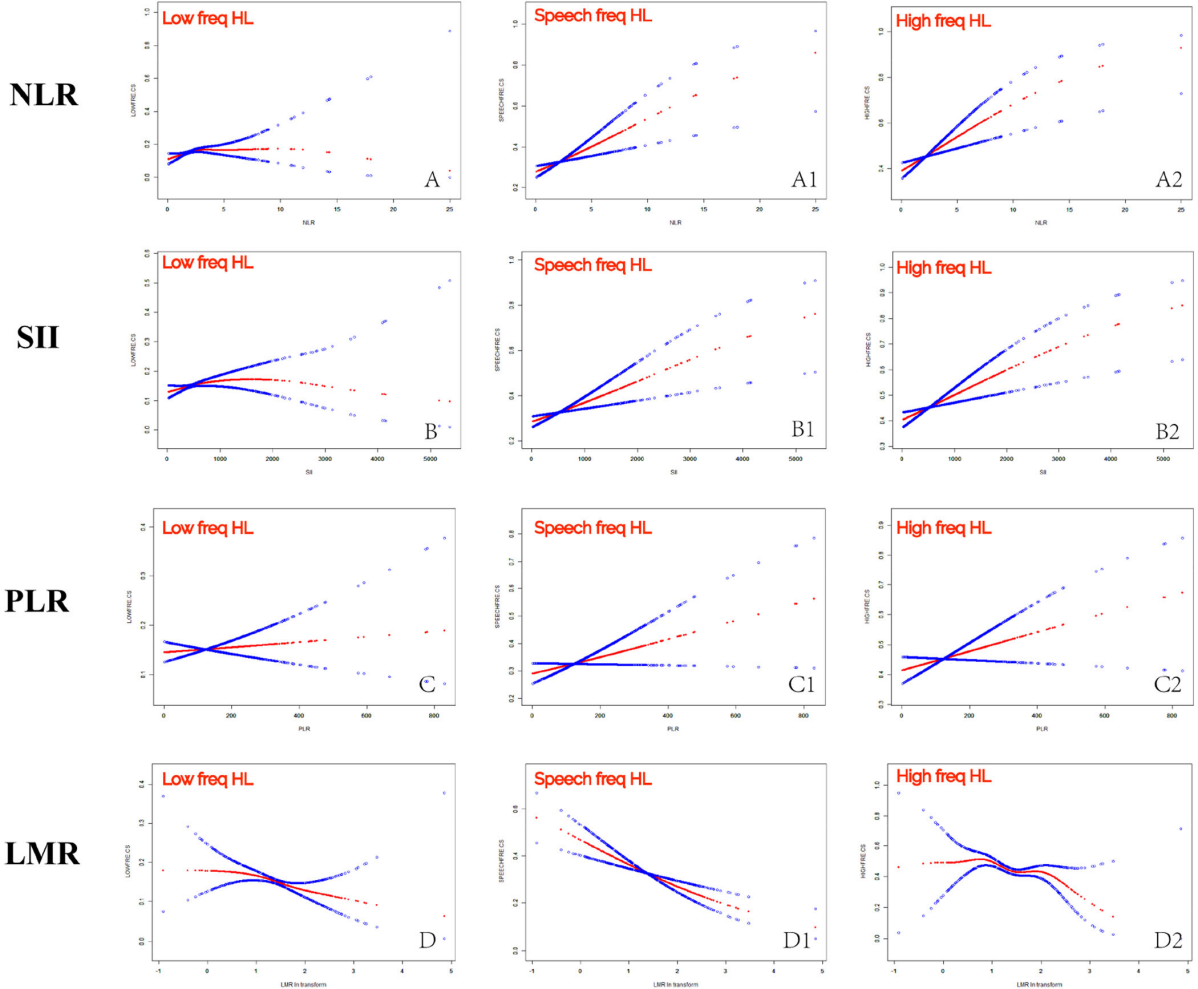
Visualization of the relationship between inflammatory index and HL. A, A1, and A2 show the relationship between NLR and low‐freq, speech‐freq, and high‐freq HL. B, B1, and B2 show the relationship between SII and low‐freq, speech‐freq, and high‐freq HL. C, C1, and C2 show the relationship between PLR and low‐freq, speech‐freq, and high‐freq HL. D, D1, and D2 show the relationship between LMR and low‐freq, speech‐freq, and high‐freq HL. The blue lines represent the fitted regression lines, and the red lines represent the 95%.

To further visualize the relationship between NLR, SII, PLR, and LMR with HL at all frequencies, smooth curve fitting was performed. In Figure 2, the positive associations of NLR and SII with the prevalence of HL at all frequencies are visualized, as well as the negative associations of LMR with the prevalence of HL at all frequencies.

The threshold saturation effect analysis results show that NLR is linearly associated with high‐frequency HL, SII is linearly associated with both low‐frequency and high‐frequency HL, and LMR is linearly associated with HL at all frequencies. However, the associations between NLR and low/speech frequencies and between SII and speech frequencies are not simple linear correlations, as indicated by a log‐likelihood ratio < 0.05, and should be interpreted with caution (Table 3).

**Table 3:** Threshold effect analysis of NLR/SII/LMR and low/speech/high‐frequency hearing loss (HL) (In‐transformed of the index).

Index	OR (95% CI), *p* value of HL		
	Low‐frequency HL	Speech‐frequency HL	High‐frequency HL
NLR			
Fitting by the standard linear mode	1.22 (1.04, 1.45) 0.0163	1.28 (1.09, 1.50) 0.0025	1.29 (1.10, 1.51) 0.0014
Fitting by the two‐piecewise linear model			
Inflection point	0.8	1.3	1.37
Index < K point	1.59 (1.19, 2.13) 0.0019	1.15 (0.96, 1.38) 0.1341	1.21 (1.02, 1.44) 0.0324
Index ≥ K point	0.90 (0.65, 1.25) 0.5200	3.44 (1.43, 8.25) 0.0057	3.50 (1.13, 10.85) 0.0300
*p* for log‐likelihood ratio	0.030	0.020	0.071
SII			
Fitting by the standard linear mode	1.17 (1.02, 1.34) 0.0273	1.20 (1.05, 1.37) 0.0078	1.21 (1.06, 1.39) 0.0045
Fitting by the two‐piecewise linear model			
Inflection point	6.14	6.71	6.73
Index < K point	1.39 (1.07, 1.81) 0.0126	1.07 (0.91, 1.26) 0.3861	1.13 (0.97, 1.32) 0.1163
Index ≥ K point	0.98 (0.76, 1.27) 0.8797	2.54 (1.37, 4.72) 0.0031	2.08 (1.07, 4.03) 0.0305
*p* for log‐likelihood ratio	0.113	0.013	0.101
LMR			
Fitting by the standard linear mode	0.78 (0.64, 0.96) 0.0173	0.65 (0.53, 0.79) < 0.0001	0.73 (0.60, 0.88) 0.0009
Fitting by the two‐piecewise linear model			
Inflection point	0.94	1.57	1.95
Index < K point	1.24 (0.68, 2.23) 0.4826	0.57 (0.44, 0.75) < 0.0001	0.78 (0.63, 0.96) 0.0214
Index ≥ K point	0.68 (0.51, 0.89) 0.0045	0.86 (0.53, 1.39) 0.5427	0.40 (0.17, 0.97) 0.0421
*p* for log‐likelihood ratio	0.104	0.213	0.155

### 3.5 Subgroup Analyses

A subgroup analysis was conducted to further investigate whether the indicators that were significantly associated with HL in the multivariate logistic regression models (NLR, SII, and LMR) exhibit differences among various subgroups and to determine how these inflammatory markers influence hearing in different populations.

**Figure 3:**
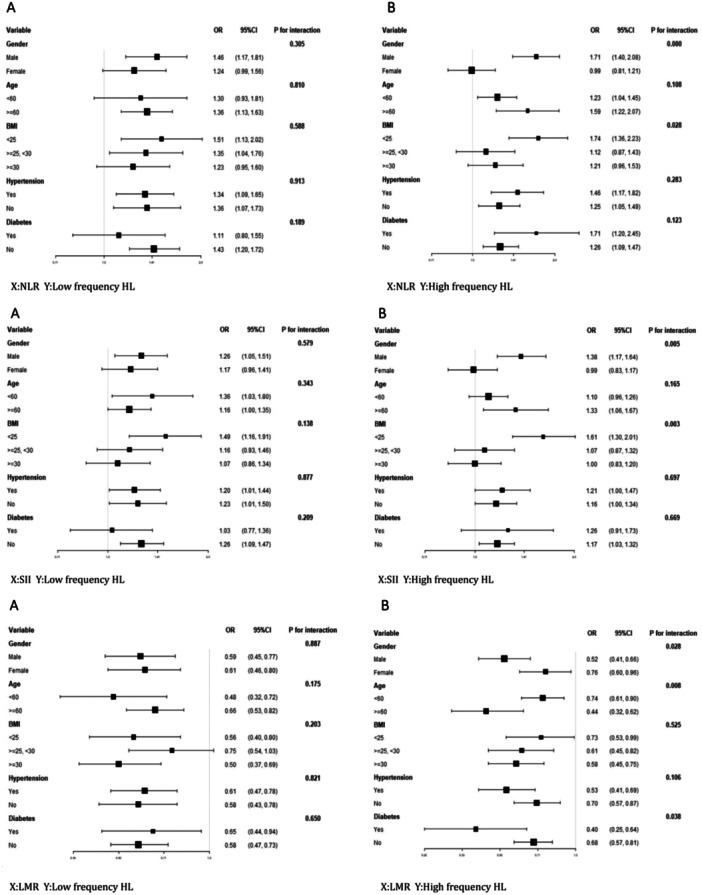
1. Interaction effects of various factors on the relationship between NLR and HL. A: Interaction effects between NLR and low‐freq HL. No interaction effects were detected among the subgroups. B: Interaction effects between NLR and high‐freq HL. Interaction effects were detected in the gender and BMI subgroups, with *p* for interaction < 0.05. 2. Interaction effects of various factors on the relationship between SII and HL. A: Interaction effects between SII and low‐freq HL. No interaction effects were detected among the subgroups. B: Interaction effects between SII and high‐freq HL. Interaction effects were detected in the gender and BMI subgroups, with *p* for interaction < 0.05. 3. Interaction effects of various factors on the relationship between LMR and HL. A: Interaction effects between LMR and low‐freq HL. No interaction effects were detected among the subgroups. B: Interaction effects were detected in the gender, age, and diabetes subgroups, with *p* for interaction < 0.05.

In Figure 3‐1, the association between NLR and low‐frequency HL showed no significant differences among various subgroups, indicating that the significant positive correlation between NLR and the prevalence of low‐frequency HL is robust and not influenced by different subgroups of gender, age, BMI, hypertension, and diabetes (Figure 3‐1A). However, interestingly, in the subgroup analysis of the association between NLR and high‐frequency HL, a significant positive correlation was observed in the male subgroup, but not in the female subgroup, with a significant interaction between groups (*p* for interaction < 0.05). Additionally, in the BMI subgroup, a significant correlation was found among participants with a normal BMI, whereas no significant correlation was observed among overweight and obese participants (BMI ≥ 25 kg/m^2^). A significant interaction between groups was also observed (*p* for interaction < 0.05) (Figure 3‐1B).

Similarly, as shown in Figure 3‐2, the association between SII and low‐frequency HL did not show significant differences among various subgroups (Figure 3‐2A). However, in the subgroup analysis of the association between SII and high‐frequency HL, a significant positive correlation was observed in the male subgroup, but not in the female subgroup, with a significant interaction between groups (*p* for interaction < 0.05). Additionally, in the BMI subgroup, a significant correlation was found among participants with a normal BMI, whereas no significant correlation was observed among overweight and obese participants (BMI ≥ 25 kg/m^2^). A significant interaction between groups was also observed (*p* for interaction < 0.05) (Figure 3‐2B).

We were surprised to find that the subgroup analysis of the association between LMR and high‐frequency HL showed significant interactions between groups in the gender, age, and BMI subgroups (*p* for interaction < 0.05). All subgroups demonstrated a significant negative correlation. However, the effect size was larger in the male subgroup, the elderly subgroup aged ≥ 60 years, and the overweight and obese subgroup with a BMI ≥ 25. Figure 3‐3 shows these findings.

## 4 Discussion

Our research thoroughly examined the relationship between the US population's systemic immune‐inflammatory biomarkers and HL. The systemic inflammatory indices were normalized using ln‐transformation. Here are several significant findings from this study. Firstly, among the four composite inflammatory markers investigated, NLR and SII were positively associated with hearing threshold shifts across all frequency categories, as well as with low‐frequency and high‐frequency HL. Conversely, LMR was negatively associated with the prevalence of HL and threshold shifts across all categorized frequencies, while PLR showed no significant correlation with HL. Secondly, there was a robust dose–response relationship between LMR and the prevalence of HL at all categorized frequencies. Thirdly, NLR and SII were identified as independent risk factors for high‐frequency HL in males and individuals with normal BMI; however, further confirmation is needed for female patients and overweight individuals. Fourthly, the correlation between LMR and HL was more pronounced in males, elderly individuals (over 60 years old), and those with diabetes, indicating population heterogeneity. Lastly, while NLR and SII were more strongly correlated with high‐frequency HL, LMR consistently showed a robust negative correlation with HL across all categorized frequencies, suggesting that LMR may be a more significant warning marker for HL.

Although age adjustments were made in the analyses, the significant age difference between no HL group (average age 30–40 years, PTA < 25 dB) and the HL group (average age over 60 years, PTA ≥ 25 dB) may still impact the results. Since age is not only a major risk factor for HL but also relates to multiple physiological and metabolic parameters, these data should be interpreted cautiously to account for potential confounding effects of age on the relationship between hearing levels and systemic inflammatory parameters.

In addition, subgroup analyses revealed heterogeneity in NLR, SII, and LMR within gender subgroups, prompting a review of the correlation between inflammation and sex hormones. Findings indicated an association between inflammatory markers and sex hormones, though the exact mechanisms underlying this relationship remain undetermined [30]. This indicates that the interactions between the immune system and the reproductive system are complex and need further investigation. Nevertheless, based on our findings, it may be suggested that greater attention be directed toward maintaining inflammatory homeostasis in the male population compared to females.

The impact of inflammation on HL development has gained significant attention. Key inflammatory markers such as CRP, TNF‐α, white blood cell and neutrophil counts, and ILs have been linked to HL. These early single inflammation markers are relevant to the correlation between inflammation and HL, which the study aims to explore. However, some studies have failed to detect a correlation between blood inflammation markers and HL [38]. This inconsistency may be due to sample size issues or the sensitivity of the chosen markers, as different markers have varying predictive values. Thus, this highlights the value of the study in exploring a range of composite inflammation markers. Given that individual inflammatory markers are easily influenced by various factors, composite inflammation markers have been developed to improve diagnostic accuracy, enhance predictive ability, and comprehensively reflect the inflammation status. Composite inflammation markers, based on routine lab tests, offer clinical value due to their convenience and accessibility. Different markers highlight various inflammatory states. While NLR [25] and PLR [39] have been studied with sudden HL, their predictive value primarily pertains to their prognosis. However, sudden HL differs from other types of HL, especially chronic, progressive loss due to aging and environmental factors. Therefore, this study uses national multi‐period data to identify more applicable, sensitive, and stable markers, enhancing clinical application.

Furthermore, although SII is a new composite inflammation marker that reflects the body's inflammation and immune status, it aligns with our research findings and has been validated in a large population using NHANES data [28]. However, in this study, we cannot conclude that it is more valuable than NLR to the prevalence of HL, despite both showing a positive correlation. Notably, SII demonstrated a more robust dose–response relationship with high‐frequency HL than NLR. This may be related to the heterogeneity in the positioning of cochlear tissue macrophages and their frequency distribution [40]. Macrophages exhibit different morphologies in the basement membrane: dendritic‐like at the apical region, amoeboid in the intermediate region, and spherical at the basal area. This suggests that they play distinct roles in the immune response of the cochlea [40, 41, 42].

Interestingly, considering the dose–response relationship, population heterogeneity, and effect size in subgroup analyses, we found that the association between the LMR and the prevalence of HL was more robust. Additionally, LMR further indicated that males, older adults, and individuals with diabetes might be at higher risk within the HL population, which has significant clinical implications and value. While there is limited research evaluating the protective effects of LMR on HL, some evidence has shown consistent beneficial effects of high levels of LMR in other conditions, including but not limited to stroke, cancer, and chronic kidney disease [14, 43, 44, 45]. Conversely, compared to the other three indicators, PLR only detected a correlation with hearing thresholds and did not show a significant association with HL. Therefore, PLR has limited predictive value as a biomarker for progressive HL. This is inconsistent with the previously observed correlation between PLR and sudden deafness in earlier studies [39]. We believe this discrepancy is due to sudden deafness being an acute inflammatory condition, which results in different bodily responses.

**Figure 4:**
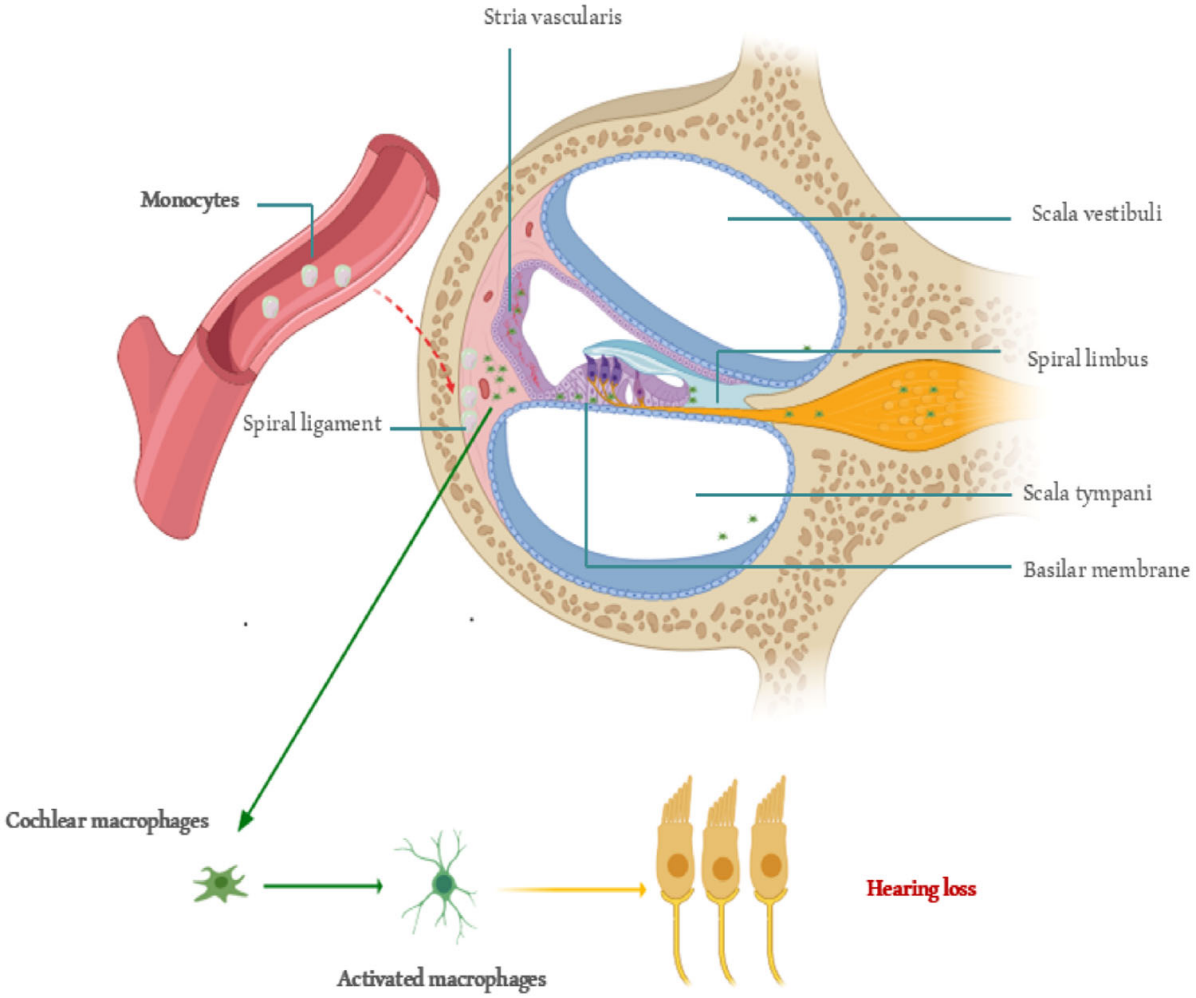
The role of cochlear macrophages in hearing loss: pathway and activation. Monocytes enter the spiral ligament through the microvascular network of the stria vascularis and differentiate into macrophages. Both transformed macrophages and resident cochlear macrophages become activated and contribute to hearing loss. The diagram shows key cochlear structures, including the stria vascularis, spiral ligament, scala vestibuli, scala tympani, spiral limbus, and basilar membrane. Macrophage activation may be a mechanism of immune‐related hearing loss.

The cochlea has traditionally been regarded as a privileged organ in the immune system, protected from the systemic immune response by the blood–brain barrier. However, recent evidence suggests that significant immune activity occurs in both normal and stressed conditions [40]. In recent years, the impact of inflammation on HL and its potential mechanisms has become a focal point in deafness research. Research has mainly concentrated on the pathogenesis of ISHL [19], noise‐induced HL [46, 47], ototoxicity from drugs (such as the chemotherapy drug cisplatin [48, 49] and aminoglycoside antibiotics [50, 51]), and age‐related HL [8, 52, 53]. Additionally, the effects of chronic diseases on HL, such as diabetes [27] and lipid metabolism disorders [54, 55], have garnered increasing attention. These findings indicate that inflammatory immune responses play a crucial role in the development of HL. It is established that inflammatory mediators will migrate to the injured tissue before macrophages. Consequently, there is a greater significance and value in monitoring changes in some inflammatory indicators in the blood at an earlier stage. The exact entry point of monocytes into the basilar membrane region remains unclear. Spiral veins and their tributaries, vascular striae [56], and spiral ligaments of the lateral wall are possible [40]. Various insults can induce cochlear macrophages to express MHC II, which activates antigen presentation. Interestingly, this activation is site‐specific, mainly occurring in the basal part of the basement membrane (high‐frequency region), where sensory cell damage is more common. The activation of the antigen‐presenting function occurs in response to sensory cell damage. This further explains why high‐frequency hearing is more susceptible to damage and less likely to recover. Thus, the physiological and anatomical bases of immunoinflammatory activation of cochlear sensory cells support the conclusion that LMR helps protect against hearing damage. LMR is important because the monocyte count in the ratio indicates how many monocytes might enter the cochlea. More monocytes lead to more cochlear macrophages acting as antigen‐presenting cells, which can damage sensory cells. Therefore, maintaining the homeostatic role of cochlear tissue‐resident macrophages and avoiding activation of circulating monocytes are key to preventing cochlear sensory cell damage (Figure 4). Our results emphasize the important role that inflammation plays in the development of HL. Since the expression of inflammatory mediators occurs before macrophage migration into injured tissues, directly inhibiting these molecules represents an effective therapeutic strategy for managing inflammation.

### 4.1 Strengths and Limitations

Our study has several strengths, including the analysis of a large, nationally representative dataset, which enhances the generalizability of our findings. By investigating multiple composite inflammatory markers (NLR, SII, LMR, and PLR), we provide a comprehensive view of their relationships with HL. We identified robust dose–response relationships and independent risk factors for high‐frequency HL, particularly in males and individuals with normal BMI. Our study also highlights the clinical significance of LMR as a potential early warning marker for HL, especially in males, older adults, and individuals with diabetes. However, this study has several limitations. First, the hearing exams covered a limited frequency range, and inflammatory biomarkers exhibit inherent variability, potentially impacting result consistency. Additionally, the lack of longitudinal data limits the ability to establish causative links between biomarkers and HL. The absence of word recognition scores may also affect a comprehensive assessment of functional hearing. Furthermore, due to inconsistencies in NHANES noise exposure data coding and high missing data rates, noise exposure was not included as a covariate, despite its known role in HL. These limitations indicate the need for further longitudinal studies to validate findings and explore actionable biomarkers for HL.

## 5 Conclusions

Our study highlights the significant role of systemic immune‐inflammatory biomarkers in HL. NLR and SII were positively associated with HL, while LMR showed a strong negative correlation, suggesting its potential as an early warning marker. We also found a dose–response relationship between LMR and HL, particularly in males, elderly individuals, and those with diabetes. However, the variability in marker sensitivity and the limited predictive value of PLR for progressive HL remain challenges. Additionally, regulating peripheral blood inflammatory factors before the activation of inner ear immune responses may be a potential target for HL treatment. Future research should focus on validating these findings in diverse populations and exploring the long‐term effects of inflammation on HL.

## Author Contributions

**Zhe Peng:** conceptualization, data curation, formal analysis, funding acquisition, investigation, methodology, project administration, supervision, writing – original draft, writing – review and editing. **Chun‐li Zhao:** data curation, formal analysis, validation, visualization, writing – review and editing. **Guo‐peng Wang:** data curation, formal analysis, writing – review and editing. **Qian Wu:** data curation, formal analysis, methodology, writing – review and editing. **Shu‐sheng Gong:** conceptualization, funding acquisition, project administration, supervision, writing – original draft, writing – review and editing.

## Ethics Statement

The studies involving human participants were reviewed and approved by the NCHS Research Ethics Review Board (ERB).

## Consent

Relevant data from participants were collected from the publicly accessible NHANES database, eliminating the need to obtain additional consent. In accordance with national legislation and institutional requirements, written informed consent for participation was not required for this study.

## Conflicts of Interest

The authors declare no conflicts of interest.

### 1 Data Availability Statement

Data from the present study are accessible at https://wwwn.cdc.gov/nchs/nhanes/Default.aspx.

## References

[1] L. M. Haile , A. U. Orji , K. M. Reavis , et al., “Hearing Loss Prevalence, Years Lived With Disability, and Hearing Aid Use in the United States From 1990 to 2019: Findings From the Global Burden of Disease Study,” Ear & Hearing 45 (2024): 257–267. 37712826 10.1097/AUD.0000000000001420 PMC10718207

[2] D. Kociszewska and S. Vlajkovic , “Age‐Related Hearing Loss: The Link Between Inflammaging, Immunosenescence, and Gut Dysbiosis,” International Journal of Molecular Sciences 23 (2022): 7348. 35806352 10.3390/ijms23137348 PMC9266910

[3] S. Chadha , K. Kamenov , and A. Cieza , “The World Report on Hearing, 2021,” Bulletin of the World Health Organization 99 (2021): 242–242A. 33953438 10.2471/BLT.21.285643 PMC8085630

[4] F. R. Lin , “Age‐Related Hearing Loss,” New England Journal of Medicine 390 (2024): 1505–1512. 38657246 10.1056/NEJMcp2306778

[5] G. Livingston , J. Huntley , A. Sommerlad , et al., “Dementia Prevention, Intervention, and Care: 2020 Report of the Lancet Commission,” Lancet 396 (2020): 413–446. 32738937 10.1016/S0140-6736(20)30367-6 PMC7392084

[6] C. S. Brown , S. D. Emmett , S. K. Robler , and D. L. Tucci , “Global Hearing Loss Prevention,” Otolaryngologic Clinics of North America 51 (2018): 575–592. 29525388 10.1016/j.otc.2018.01.006

[7] S. D. Nash , K. J. Cruickshanks , W. Zhan , et al., “Long‐Term Assessment of Systemic Inflammation and the Cumulative Incidence of Age‐Related Hearing Impairment in the Epidemiology of Hearing Loss Study,” Journals of Gerontology: Series A 69 (2014): 207–214. 10.1093/gerona/glt075 PMC4038239 23739996

[8] N. Watson , B. Ding , X. Zhu , and R. D. Frisina , “Chronic Inflammation— Inflammaging—in the Ageing Cochlea: A Novel Target for Future Presbycusis Therapy,” Ageing Research Reviews 40 (2017): 142–148. 29017893 10.1016/j.arr.2017.10.002 PMC5675822

[9] P. Bazard , J. Pineros , R. D. Frisina , et al., “Cochlear Inflammaging in Relation to Ion Channels and Mitochondrial Functions,” Cells 10 (2021): 2761. 34685743 10.3390/cells10102761 PMC8534887

[10] K. N. Prasad and S. C. Bondy , “Increased Oxidative Stress, Inflammation, and Glutamate: Potential Preventive and Therapeutic Targets for Hearing Disorders,” Mechanisms of Ageing and Development 185 (2020): 111191. 31765645 10.1016/j.mad.2019.111191

[11] A. Maraslioglu‐Sperber , F. Blanc , and S. Heller , “Murine Cochlear Damage Models in the Context of Hair Cell Regeneration Research,” Hearing Research 447 (2024): 109021. 38703432 10.1016/j.heares.2024.109021

[12] A. Chavarria and J. Alcocer‐Varela , “Is Damage in Central Nervous System due to Inflammation,” Autoimmunity Reviews 3 (2004): 251–260. 15246020 10.1016/j.autrev.2003.09.006

[13] W. Cheng , X. Bu , C. Xu , et al., “Higher Systemic Immune‐Inflammation Index and Systemic Inflammation Response Index Levels Are Associated With Stroke Prevalence in the Asthmatic Population: A Cross‐Sectional Analysis of the NHANES 1999‐2018,” Frontiers in Immunology 14 (2023): 1191130. 37600830 10.3389/fimmu.2023.1191130 PMC10436559

[14] Y. Chen , Y. Nie , J. Wu , et al., “Association Between Systemic Inflammatory Indicators With the Survival of Chronic Kidney Disease: A Prospective Study Based on NHANES,” Frontiers in immunology 15 (2024): 1365591. 38650947 10.3389/fimmu.2024.1365591 PMC11033417

[15] J. Li , X. Wang , W. Jia , et al., “Association of the Systemic Immuno‐Inflammation Index, Neutrophil‐to‐Lymphocyte Ratio, and Platelet‐to‐Lymphocyte Ratio With Diabetic Microvascular Complications,” Frontiers in Endocrinology 15 (2024): 1367376. 38660516 10.3389/fendo.2024.1367376 PMC11039910

[16] T. Zhou , M. Chen , Z. Yuan , et al., “Inflammatory Markers and the Risk of Idiopathic Sudden Sensorineural Hearing Loss: A Mendelian Randomization Study,” Frontiers in Neurology 14 (2023): 1111255. 36908593 10.3389/fneur.2023.1111255 PMC9992207

[17] J. Wang , M. Liu , V. Sung , et al., “Does Inflammation Mediate the Association Between Obesity and Hearing Status in Mid‐Childhood and Mid‐Life,” International Journal of Obesity 46 (2022): 1188–1195. 35220416 10.1038/s41366-022-01080-9

[18] M. Fujioka , S. Kanzaki , H. J. Okano , M. Masuda , K. Ogawa , and H. Okano , “Proinflammatory Cytokines Expression in Noise‐Induced Damaged Cochlea,” Journal of Neuroscience Research 83 (2006): 575–583. 16429448 10.1002/jnr.20764

[19] M. Masuda , S. Kanzaki , S. Minami , et al., “Correlations of Inflammatory Biomarkers With the Onset and Prognosis of Idiopathic Sudden Sensorineural Hearing Loss,” Otology & Neurotology 33 (2012): 1142–1150. 22872174 10.1097/MAO.0b013e3182635417

[20] X. Tan , J. Pan , J. Cai , et al., “Relevant Research of Inflammatory Cytokines Spectrum in Peripheral Blood of Sudden Hearing Loss,” Laryngoscope 134 (2024): 3293–3301. 38193513 10.1002/lary.31276

[21] C. A. Verschuur , A. Dowell , H. E. Syddall , et al., “Markers of Inflammatory Status Are Associated With Hearing Threshold in Older People: Findings From the Hertfordshire Ageing Study,” Age and Ageing 41 (2012): 92–97. 22086966 10.1093/ageing/afr140

[22] C. Lassale , P. Vullo , D. Cadar , G. D. Batty , A. Steptoe , and P. Zaninotto , “Association of Inflammatory Markers With Hearing Impairment: The English Longitudinal Study of Ageing,” Brain, Behavior, and Immunity 83 (2020): 112–119. 31562886 10.1016/j.bbi.2019.09.020 PMC6906240

[23] S. Parekh and T. Kaur , “Cochlear Inflammaging: Cellular and Molecular Players of the Innate and Adaptive Immune System in Age‐Related Hearing Loss,” Frontiers in Neurology 14 (2023): 1308823. 38073631 10.3389/fneur.2023.1308823 PMC10702987

[24] R. Ha , B. W. Lim , D. H. Kim , J. W. Park , C. H. Cho , and J. H. Lee , “Predictive Values of Neutrophil to Lymphocyte Ratio (NLR), Platelet to Lymphocyte Ratio (PLR), and Other Prognostic Factors in Pediatric Idiopathic Sudden Sensorineural Hearing Loss,” International Journal of Pediatric Otorhinolaryngology 120 (2019): 134–139. 30784810 10.1016/j.ijporl.2019.02.023

[25] J. G. Doo , D. Kim , Y. Kim , et al., “Biomarkers Suggesting Favorable Prognostic Outcomes in Sudden Sensorineural Hearing Loss,” International Journal of Molecular Sciences 21 (2020): 7248. 33008090 10.3390/ijms21197248 PMC7583026

[26] S. Ulu , M. S. Ulu , A. Bucak , A. Ahsen , F. Yucedag , and A. Aycicek , “Neutrophil‐to‐Lymphocyte Ratio as a New, Quick, and Reliable Indicator for Predicting Diagnosis and Prognosis of Idiopathic Sudden Sensorineural Hearing Loss,” Otology & Neurotology 34 (2013): 1400–1404. 23988996 10.1097/MAO.0b013e31829b57df

[27] S. Ulu , A. Bucak , M. S. Ulu , et al., “Neutrophil‐Lymphocyte Ratio as a New Predictive and Prognostic Factor at the Hearing Loss of Diabetic Patients,” European Archives of Oto‐Rhino‐Laryngology 271 (2014): 2681–2686. 24121821 10.1007/s00405-013-2734-3

[28] T. Zhou , J. Mao , P. Zhu , X. Yu , and X. Yang , “Association Between the Systemic Immuno‐Inflammation Index and Hearing Loss: Result From NHANES 2009‐2018,” Frontiers in Neurology 15 (2024): 1369492. 38715688 10.3389/fneur.2024.1369492 PMC11074419

[29] Ş. Ulu , A. Kınar , A. Bucak , and M. Özdemir , “Systemic Immune Inflammatory Index of Patients With Idiopathic Sudden Sensorineural Hearing Loss: Comparison of NLR and PRL Values,” Ear, Nose, & Throat Journal 100 (2021): 726–730. 10.1177/0145561320924312 32396031

[30] Centers for Disease Control and Prevention (CDC) , The National Health and Nutritional Examination Survey (NHANES) Analytic and Reporting Guidelines (CDC, 2006).

[31] B. Hu , X. R. Yang , Y. Xu , et al., “Systemic Immune‐Inflammation Index Predicts Prognosis of Patients After Curative Resection for Hepatocellular Carcinoma,” Clinical Cancer Research 20 (2014): 6212–6222. 25271081 10.1158/1078-0432.CCR-14-0442

[32] J. Y. Pan , Y. Chen , Z. H. Lin , B. Lv , L. Chen , and S. Y. Feng , “Association Between Triglyceride‐Glucose Index and Hearing Threshold Shifts of Adults in the United States: National Health and Nutrition Examination Survey, 2015‐2016,” Journal of Multidisciplinary Healthcare 17 (2024): 1791–1801, 10.2147/JMDH.S454678. 38686130 PMC11056606

[33] Y. Fu , W. Chen , and Y. Liu , “The Association Between Ultra‐Processed Food Intake and Age‐Related Hearing Loss: A Cross‐Sectional Study,” BMC Geriatrics 24 (2024): 450, 10.1186/s12877-024-04935-0. 38783172 PMC11118724

[34] H. J. Hoffman , R. A. Dobie , K. G. Losonczy , C. L. Themann , and G. A. Flamme , “Declining Prevalence of Hearing Loss in US Adults Aged 20 to 69 Years,” JAMA Otolaryngology—Head & Neck Surgery 143 (2017): 274–285, 10.1001/jamaoto.2016.3527. 27978564 PMC5576493

[35] F. Scinicariello and M. C. Buser , “Association of Iodine Deficiency With Hearing Impairment in US Adolescents Aged 12 to 19 Years: Analysis of NHANES 2007‐2010 Data,” JAMA Otolaryngology—Head & Neck Surgery 144 (2018): 644–645, 10.1001/jamaoto.2018.0651. 29879267 PMC6145780

[36] B. Szeto , C. Valentini , and A. K. Lalwani , “Low Vitamin D Status Is Associated With Hearing Loss in the Elderly: A Cross‐Sectional Study,” American Journal of Clinical Nutrition 113 (2021): 456–466, 10.1093/ajcn/nqaa310. 33247302

[37] G. Zipf , M. Chiappa , K. S. Porter , Y. Ostchega , B. G. Lewis , and J. Dostal , “National Health and Nutrition Examination Survey: Plan and Operations, 1999‐2010,” Vital and Health Statistics. Ser. 1: Programs and Collection Procedures 1 (2013): 1–37. 25078429

[38] S. Gupta , S. G. Curhan , and G. C. Curhan , “Biomarkers of Systemic Inflammation and Risk of Incident Hearing Loss,” Ear & Hearing 40 (2019): 981–989. 30399011 10.1097/AUD.0000000000000678 PMC6500774

[39] W. Xie , N. Karpeta , B. Tong , et al., “Etiological Analysis of Patients With Sudden Sensorineural Hearing Loss: A Prospective Case‐Control Study,” Scientific Reports 13 (2023): 5221. 36997587 10.1038/s41598-023-32085-7 PMC10063564

[40] W. Yang , R. R. Vethanayagam , Y. Dong , Q. Cai , and B. H. Hu , “Activation of the Antigen Presentation Function of Mononuclear Phagocyte Populations Associated With the Basilar Membrane of the Cochlea After Acoustic Overstimulation,” Neuroscience 303 (2015): 1–15. 26102003 10.1016/j.neuroscience.2015.05.081 PMC4532582

[41] S. Hashimoto , P. Billings , J. P. Harris , G. S. Firestein , and E. M. Keithley , “Innate Immunity Contributes to Cochlear Adaptive Immune Responses,” Audiology and Neurotology 10 (2005): 35–43. 15567913 10.1159/000082306

[42] Y. C. Liu and K. Xu , “Macrophage‐Related Immune Responses in Inner Ear: A Potential Therapeutic Target for Sensorineural Hearing Loss,” Frontiers in Neuroscience 17 (2024): 1339134. 38274500 10.3389/fnins.2023.1339134 PMC10808290

[43] P. Gong , Y. Liu , Y. Gong , et al., “The Association of Neutrophil to Lymphocyte Ratio, Platelet to Lymphocyte Ratio, and Lymphocyte to Monocyte Ratio With Post‐Thrombolysis Early Neurological Outcomes in Patients With Acute Ischemic Stroke,” Journal of Neuroinflammation 18 (2021): 51. 33610168 10.1186/s12974-021-02090-6 PMC7896410

[44] T. F. Nishijima , H. B. Muss , S. S. Shachar , K. Tamura , and Y. Takamatsu , “Prognostic Value of Lymphocyte‐to‐Monocyte Ratio in Patients With Solid Tumors: A Systematic Review and Meta‐Analysis,” Cancer Treatment Reviews 41 (2015): 971–978. 26481060 10.1016/j.ctrv.2015.10.003

[45] D. Tan , Y. Fu , W. Tong , and F. Li , “Prognostic Significance of Lymphocyte to Monocyte Ratio in Colorectal Cancer: A Meta‐Analysis,” International Journal of Surgery 55 (2018): 128–138. 29807167 10.1016/j.ijsu.2018.05.030

[46] W. J. T. Tan , P. R. Thorne , and S. M. Vlajkovic , “Characterisation of Cochlear Inflammation in Mice Following Acute and Chronic Noise Exposure,” Histochemistry and Cell Biology 146 (2016): 219–230. 27109494 10.1007/s00418-016-1436-5

[47] D. Chen , G. Jia , Y. Zhang , et al., “Sox2 Overexpression Alleviates Noise‐Induced Hearing Loss by Inhibiting Inflammation‐Related Hair Cell Apoptosis,” Journal of Neuroinflammation 19 (2022): 59. 35227273 10.1186/s12974-022-02414-0 PMC8883703

[48] E. Gentilin , E. Simoni , M. Candito , D. Cazzador , and L. Astolfi , “Cisplatin‐Induced Ototoxicity: Updates on Molecular Targets,” Trends in Molecular Medicine 25 (2019): 1123–1132. 31473143 10.1016/j.molmed.2019.08.002

[49] X. Qiao , W. Li , Z. Zheng , et al., “Inhibition of the HMGB1/RAGE Axis Protects Against Cisplatin‐Induced Ototoxicity via Suppression of Inflammation and Oxidative Stress,” International Journal of Biological Sciences 20 (2024): 784–800. 38169643 10.7150/ijbs.82003 PMC10758089

[50] M. Jiang , F. Taghizadeh , and P. S. Steyger , “Potential Mechanisms Underlying Inflammation‐Enhanced Aminoglycoside‐Induced Cochleotoxicity,” Frontiers in Cellular Neuroscience 11 (2017): 362. 29209174 10.3389/fncel.2017.00362 PMC5702304

[51] M. Jiang , H. Li , A. Johnson , et al., “Inflammation Up‐Regulates Cochlear Expression of TRPV1 to Potentiate Drug‐Induced Hearing Loss,” Science Advances 5 (2019): eaaw1836. 31328162 10.1126/sciadv.aaw1836 PMC6636990

[52] B. J. Seicol , S. Lin , and R. Xie , “Age‐Related Hearing Loss Is Accompanied by Chronic Inflammation in the Cochlea and the Cochlear Nucleus,” Frontiers in Aging Neuroscience 14 (2022): 846804. 35418849 10.3389/fnagi.2022.846804 PMC8995794

[53] J. Menardo , Y. Tang , S. Ladrech , et al., “Oxidative Stress, Inflammation, and Autophagic Stress as the Key Mechanisms of Premature Age‐Related Hearing Loss in SAMP8 Mouse Cochlea,” Antioxidants & Redox Signaling 16 (2012): 263–274. 21923553 10.1089/ars.2011.4037

[54] J. Chan , R. Telang , D. Kociszewska , P. R. Thorne , and S. M. Vlajkovic , “A High‐Fat Diet Induces Low‐Grade Cochlear Inflammation in CD‐1 Mice,” International Journal of Molecular Sciences 23 (2022): 5179. 35563572 10.3390/ijms23095179 PMC9101486

[55] D. Kociszewska , J. Chan , P. R. Thorne , and S. M. Vlajkovic , “The Link Between Gut Dysbiosis Caused by a High‐Fat Diet and Hearing Loss,” International Journal of Molecular Sciences 22 (2021): 13177. 34947974 10.3390/ijms222413177 PMC8708400

[56] J. P. Harris , S. Fukuda , and E. M. Keithley , “Spiral Modiolar Vein: Its Importance in Inner Ear Inflammation,” Acta Oto‐Laryngologica 110 (1990): 357–364. 2284910 10.3109/00016489009107455

